# Supplementary material for: Inherent strain and kinetic coupling determine the kinetics of ammonia synthesis over Ru nanoparticles
Source: Nat Commun. 2025 Feb 13;16:1625. doi: 10.1038/s41467-025-56765-2 (PMC11825680; doi:10.1038/s41467-025-56765-2)
Supplement: Supplementary file 1 — Supplementary Information [file 41467_2025_56765_MOESM1_ESM.pdf]

Supplementary Information: Inherent strain and kinetic coupling determine the kinetics of ammonia synthesis over Ru nanoparticles

Yuqi Yang[1,*] Anders Hellman[1,*] and Henrik Grönbeck[1*]

[1] *Department of Physics and Competence Centre for Catalysis, Chalmers University of Technology, SE-41296 Göteborg, Sweden*

E-mail: yuqiy@chalmers.se; ahell@chalmers.se; ghj@chalmers.se

Supplementary Methods

DFT calculations

Bulk Ru in the hcp structure was considered using a (16 × 16 × 16) k-point grid. The lattice constants were calculated to be a = 2.72 Å and c = 4.30 Å, respectively, which are in good agreement with the experimental results of a = 2.70 Å and c = 4.28 Å, respectively.[1] Supplementary Figure 7 shows atomic models of the considered surface systems. Ru(0001) was described by a (3 × 6) surface cell. Surface models of a step and an edge on Ru(0001) were constructed from the pristine Ru(0001) surface. Ru($10\bar{1}0$) and Ru($10\bar{1}1$) sites were described by (3 × 3) surface cells. Ru($10\bar{1}2$) sites were described by a (3 × 2) surface cell and Ru($2\bar{1}\bar{1}2$) were described by a (2 × 2) surface cell. All Ru slabs used in DFT calculations were built with four atomic layers, where the two bottom layers were kept fixed whereas the two top layers and adsorbates were allowed to relaxed. The structures and transition states were optimized until all forces were less than 0.03 eV/Å, using a (4 × 4 × 1) k-point grid on the slabs and a vacuum layer of 15 Å to separate the periodic images. The transition states (TS) were located by the Climbing-Image Nudged Elastic Band (CINEB) method from the VTST tools.[2] The transition states were confirmed by vibrational analysis within the harmonic approximation showing a single imaginary frequency along the reaction coordinate.

All the reaction energies in the kinetic simulations were zero-point energy correlated. Vibrational analysis is performed within the harmonic approximation using finite differences.

$$\Delta E^{ZPE} = \sum_i \frac{1}{2} h\nu_i \tag{1}$$

Where h is the Planck constant, and v_i are the computed real frequencies.

The entropy of gas S^{gas} was obtained by using the Shomate equation with parameters taken from the NIST chemistry web book.[3] The entropy of surface species S^{ads} is evaluated

in the harmonic approximation,

$$S^{ads} = k_B \sum_{i=1}^{Modes} [\frac{x_i}{e^{x_i} - 1} - ln(1 - e^{-x_i})] \quad (2)$$

$$x_i = \frac{h\mu_i}{k_B T} \quad (3)$$

Where k_B is the Boltzmann constant. It is known that for the soft modes with low frequencies, the anharmonic effects are important and the harmonic approach often fails numerically.[4] Thus, the imaginary and the low frequency modes with vibration lower than 100 cm^{-1} are set to 100 cm^{-1}. The results of ZPE and entropy correlations for each surface species and transition states at 673 K are shown in Supplementary Table 17.

Strain effect on Ru surfaces and nanoparticles

The strain effect on reaction energy is represented with respect to the unstrained surfaces.[5] The changes of reaction energies and energy barriers by strain are added into the description of each elementary reaction:

$$E_a^{strain} = E_a + \alpha \Delta s \quad (4)$$

$$\Delta E^{strain} = \Delta E + \beta \Delta s \quad (5)$$

Where α and β are the parameters fitted by DFT calculations on Ru(0001) and stepped Ru(0001) surfaces with the tensile and compressive strain of 4%, as shown in Supplementary Table 18.

To verify that the dependence on strain obtained for the Ru surface models is valid also for Ru NPs, we compare explicit calculations for the N-atom binding energy on strained Ru NP (ΔE_N^{strain}) with results for respect to that on unstrained Ru NP (ΔE_N). The Ru_{318} was considered in a (30 × 30 × 30) Å box. We calculated first the optimal structure of unstrained (Ru_{318} with every atom relaxed in three directions. Based on the structure of unstrained

Ru_{318}, we homogeneously strained the NP by +4% and -4%, respectively. The N-atom binding energies on the Ru(0001) facet of Ru_{318} NP are compared with the results for the extended Ru(0001) surface (Supplementary Figure 9). The trends of $(\Delta E_N^{strain} - \Delta E_N)$ on Ru nanoparticle and Ru surface are consistent, which verifies that the the strain dependence on the nanoparticles can be described by models developed for the extended surfaces.

Adsorbate-adsorbate interactions

The adsorbate-adsorbate interactions between surface intermediates are treated by a lattice based cluster expansion approach. In Ru-based ammonia synthesis reaction, N and NH can be assumed to be the major surface species. In Supplementary Figure 10, the nearest neighbor sites (1NN) adsorbate-adsorbate interactions of N-N, NH-NH, N-NH are fitted with configurations of different coverages calculated using $p(\sqrt{3} \times \sqrt{3})$, $p(2 \times \sqrt{3})$, $p(2 \times 2)$ and $p(3 \times 3)$ Ru(0001) surface cells. The adsorbate-adsorbate interactions between N/NH with other minor surface species (NH_2, NH_3 and N_2) are calculated by configurations using a $p(3 \times 3)$ Ru(0001) surface cell. Moreover, the adsorbate-adsorbate interactions between minor surface species are calculated using the Berthelot mixing rule,

$$\varepsilon_{ij} = \sqrt{\varepsilon_{ii}\varepsilon_{jj}} \tag{6}$$

Here ε_{ij} is the pairwise interaction between adsorbates i and j and ε_{ii} and ε_{jj} are the self-interactions of adsorbates i and j. Supplementary Table 21 shows the adsorbate-adsorbate interactions.

The influence of adsorbate-adsorbate interactions on the transition state of the N_2 dissociation step with N atom (ε_{N*-TS}) is calculated on Ru(0001) and stepped Ru(0001) surfaces (see Supplementary Figure 11). The average of ε_{N*-TS} on different configurations is calculated to be 0.31 eV, which is consistent with previous work.[6] For the NH_x hydrogenation steps, the barriers are scaled with the adsorbate-adsorbate interactions via the universal

linear scaling relations with respect to reaction energies.[7]

Scaling factor $\kappa(T)$

In the present work, the scaling factor $\kappa(T)$ for N_2 dissociation step is obtained from experiments.[8] Experimentally, the sticking coefficient of N_2 dissociative adsorption step has been measured on Ru(0001) and stepped Ru(0001).[8] According to collision theory, the rate constant (k) for N_2 adsorption is:

$$k = \frac{A\,p\,S_0(T)}{\sqrt{2\pi m k_B T}} \tag{7}$$

Here, A is the area of site, p is the N_2 pressure and $S_0(T)$ is the sticking coefficient. By fitting the temperature dependent rate constants to experiments[8] (Supplementary Figure 12), values of the scaling factor $\kappa(T)$ in the rate equation in the main text can be obtained for Ru(0001) and a stepped Ru(0001) surface. The parameters used in the calculation of κ at 673 K is shown in Supplementary Table 22. In this work, the average values of $\kappa(T)$ on Ru(0001) and a stepped Ru(0001) surface are used in kMC simulations.

DRC analysis in kMC simulations

The sensitivity analysis of TOF on kinetic parameters in the kMC simulations is based on a degree of Rate Control (DRC) analysis.[9,10] The DRC for elementary reaction i is calculated by:

$$DRC = [\frac{\partial ln(TOF)}{\partial(\Delta G_i^{act}/k_BT)}]_{\Delta G_j^{act}, j\neq i} \tag{8}$$

Where ΔG_i^{act} is the Gibbs free energy barrier of elementary reaction i. In the kMC simulations, the energies of the transition states are varied by ± 0.05 and ± 0.10 eV, and a five-point linear fitting was employed to determine the DRC.

Supplementary Discussion

Uncertainty analysis in the scaling relations

To assess the uncertainty in adsorption energies and activation barriers of N_2 dissociation and NH_x hydrogenation, we performed a sensitivity analysis on the slope of the scaling relations of N atom, NH, NH_2 and H atom on the Ru_{780} NP. As the activation barriers are computed relative to the reaction energies (with scaling relations), the sensitivity analysis on linear scaling relations also captures the uncertainty in activation barriers.

In this analysis, we first changed the slope of the scaling relations for N atom, NH, NH_2 and H atom by ±10% and ±20%, while keeping other energy parameters constant. The different cases are shown in Supplementary Figure 13. Moreover, considering that the scaling relations for the adsorption energies of N, NH, NH_2 and NH_3 are correlated,[11] we simultaneously adjusted the slopes in the scaling relations by ±10% and ±20%, to investigate the combined effects of NH_x surface species. The results of sensitivity analysis on the TOF of Ru_{780} NP are shown in Supplementary Table 23. The relative TOF difference (ΔTOF) is calculated according to Supplementary Equation (9),

$$\Delta TOF = \frac{TOF - TOF_{unchanged}}{TOF_{unchanged}} \times 100 \tag{9}$$

Where $TOF_{unchanged}$ is the TOF calculated with the original slope. The sensitivity analysis shows that the slope variations in the linear scaling relations on each surface species have small effects on the TOF. The small effects can be attributed to the wide distribution of sites (many different GCNs) on Ru NPs. For example, an increase in the slope of N adsorption energy raises the N_2 dissociation activation barrier at high GCN sites but reduces it at low GCN sites. This site-specific compensation effect reduces the overall influence of energy changes on the reaction kinetics.

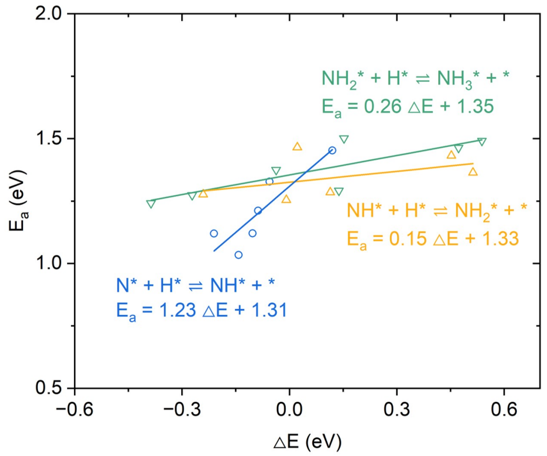

Supplementary Figure 1: Energy barriers of the NH_x hydrogenation steps versus reaction energies.

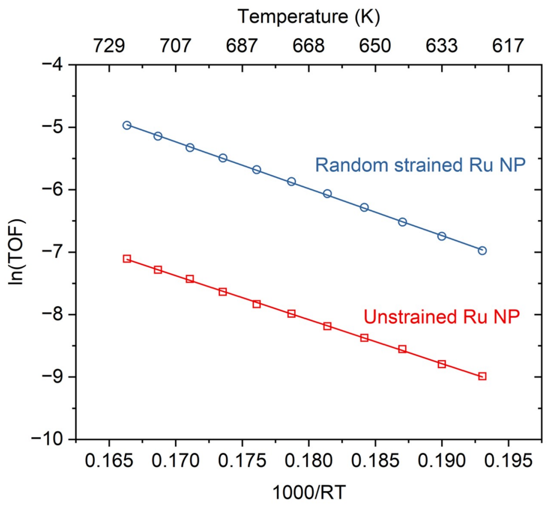

Supplementary Figure 2: Arrhenius plots on unstrained and randomly ($\sigma = 0.05$) strained Ru_{780}.

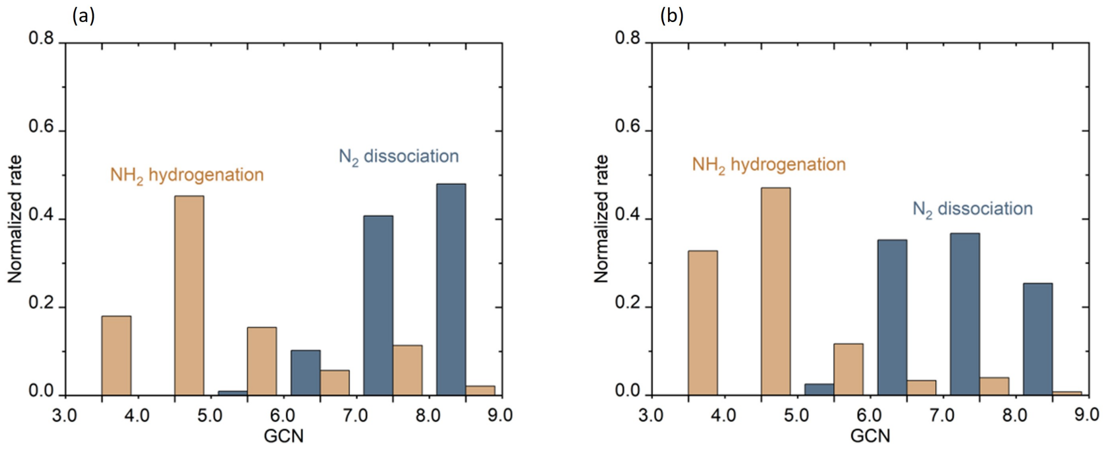

Supplementary Figure 3: Normalized rate of N_2 dissociation and NH_3 hydrogenation steps versus GCN on (a) +4% homogeneous strained and (b) randomly ($\sigma = 0.05$) strained Ru Ru_{780}.

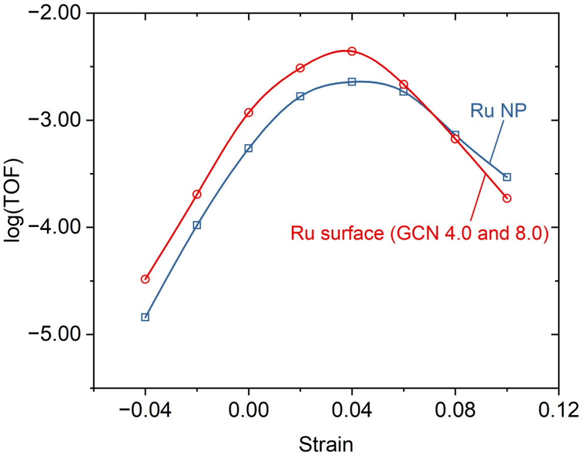

Supplementary Figure 4: TOF versus strain on Ru_{780} and simplified model with GCN 4.0 and 8.0

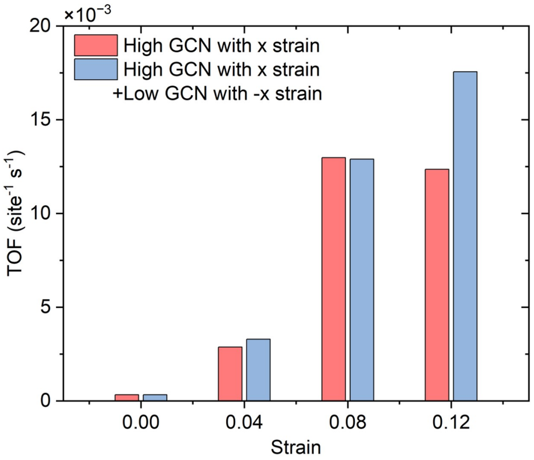

Supplementary Figure 5: TOF of Ru_{780} versus strain. The red bars show the case when applying the tensile strain on high GCN sites, the blue bars show the case when applying the tensile strain on high GCN sites and compressive strain on low GCN sites.

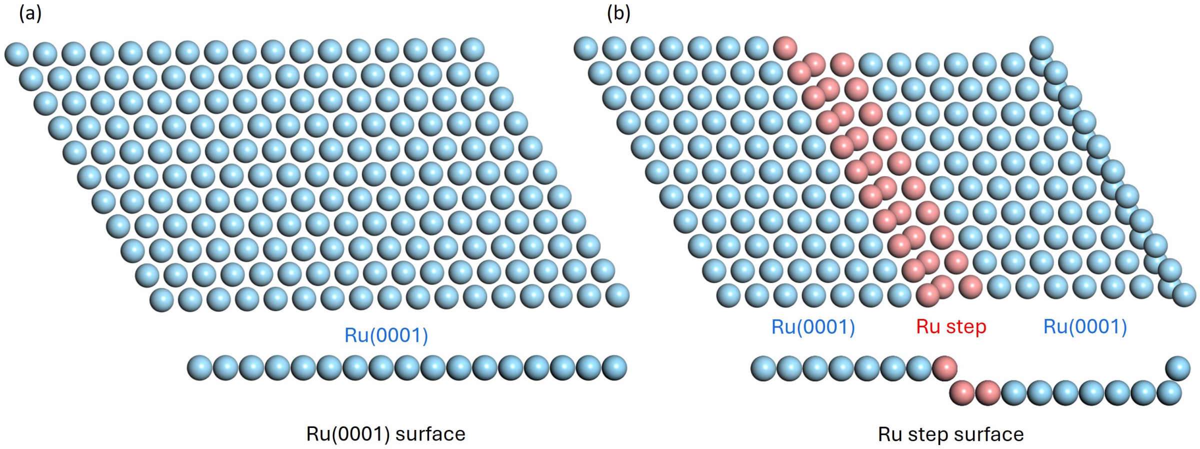

Supplementary Figure 6: Surface models of (a) Ru(0001) and (b) a Ru(0001) surface with a step.

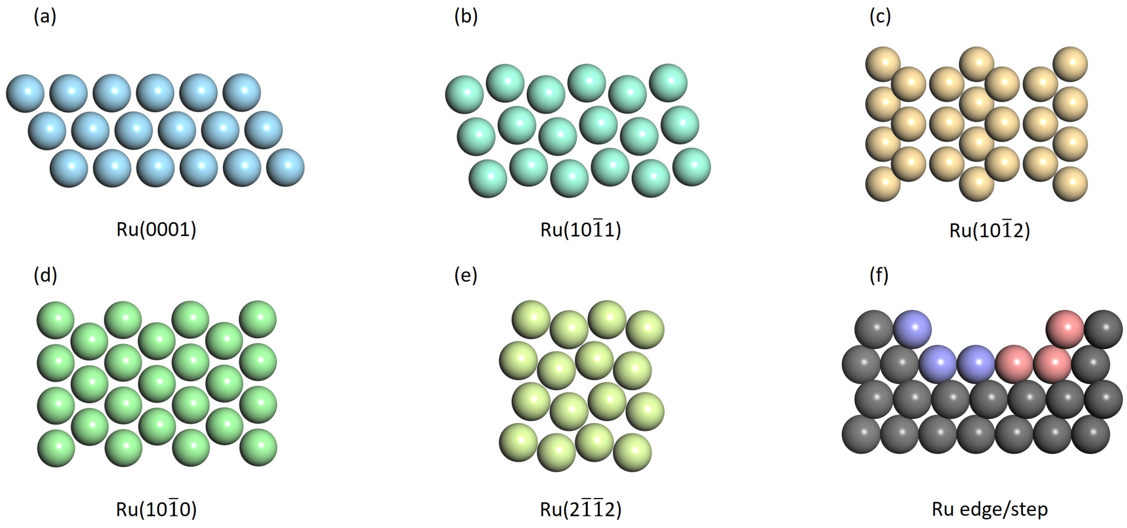

Supplementary Figure 7: Top view of (a) Ru(0001), (b) Ru(10$\bar{1}$1), (c) Ru(10$\bar{1}$2), (d) Ru(10$\bar{1}$0), (e) Ru(2$\bar{1}\bar{1}$2). (f) Side view of B_5 (blue ball) and A_5 (red ball) step/edge sites.

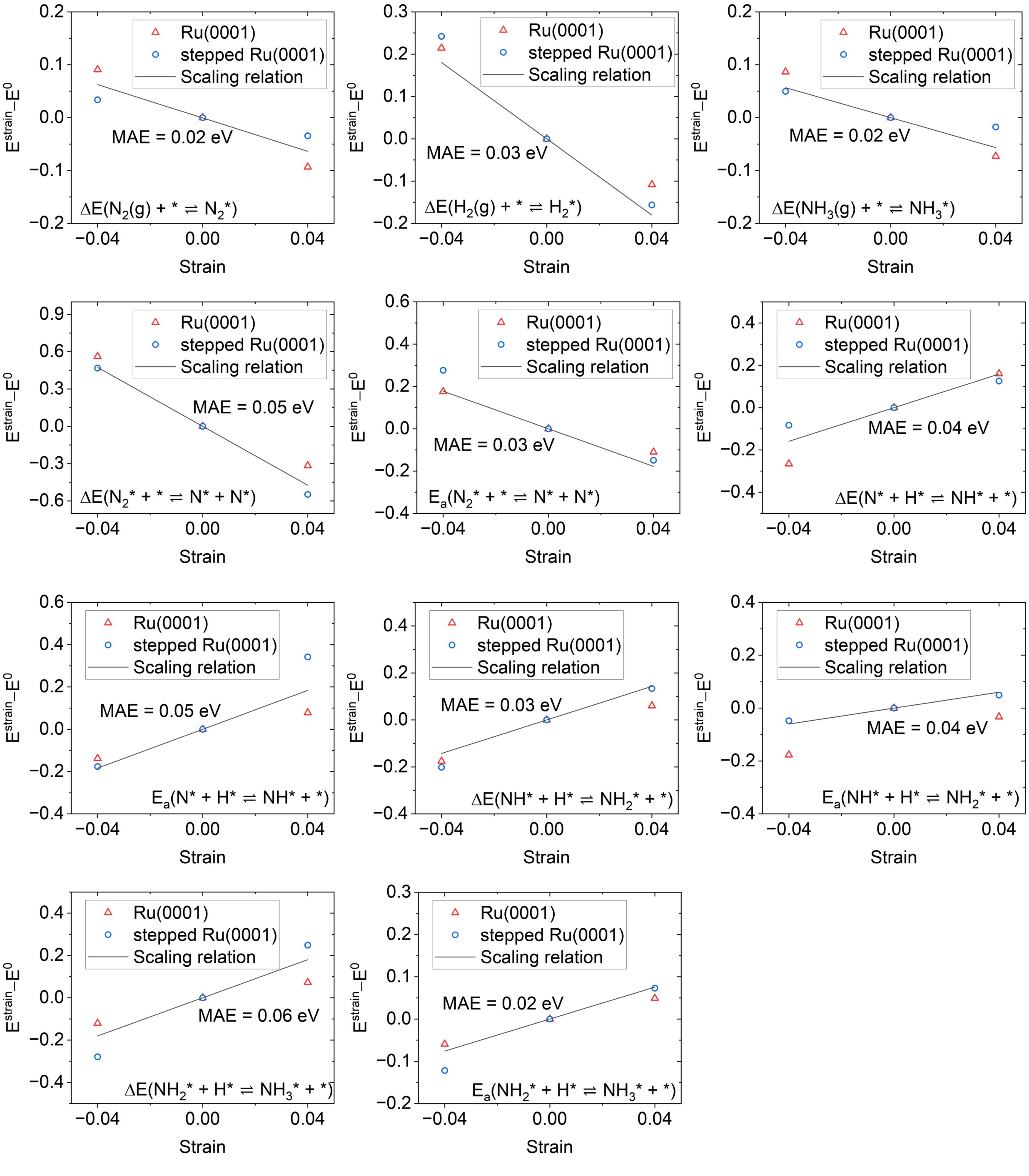

Supplementary Figure 8: Scaling relations of reaction energies and barriers with respect to strain.

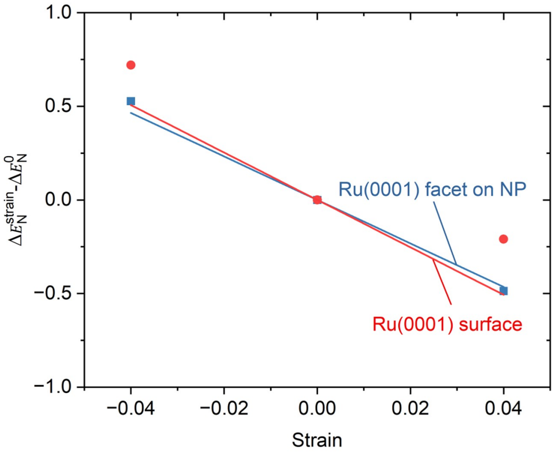

Supplementary Figure 9: N-atom binding energy difference with respect to strain calculated on the Ru(0001) facet of the Ru_{380} NP and on the extended Ru(0001) surface. The solid lines are linear fits to the data (filled symbols) with the constrain that the difference is zero at zero strain.

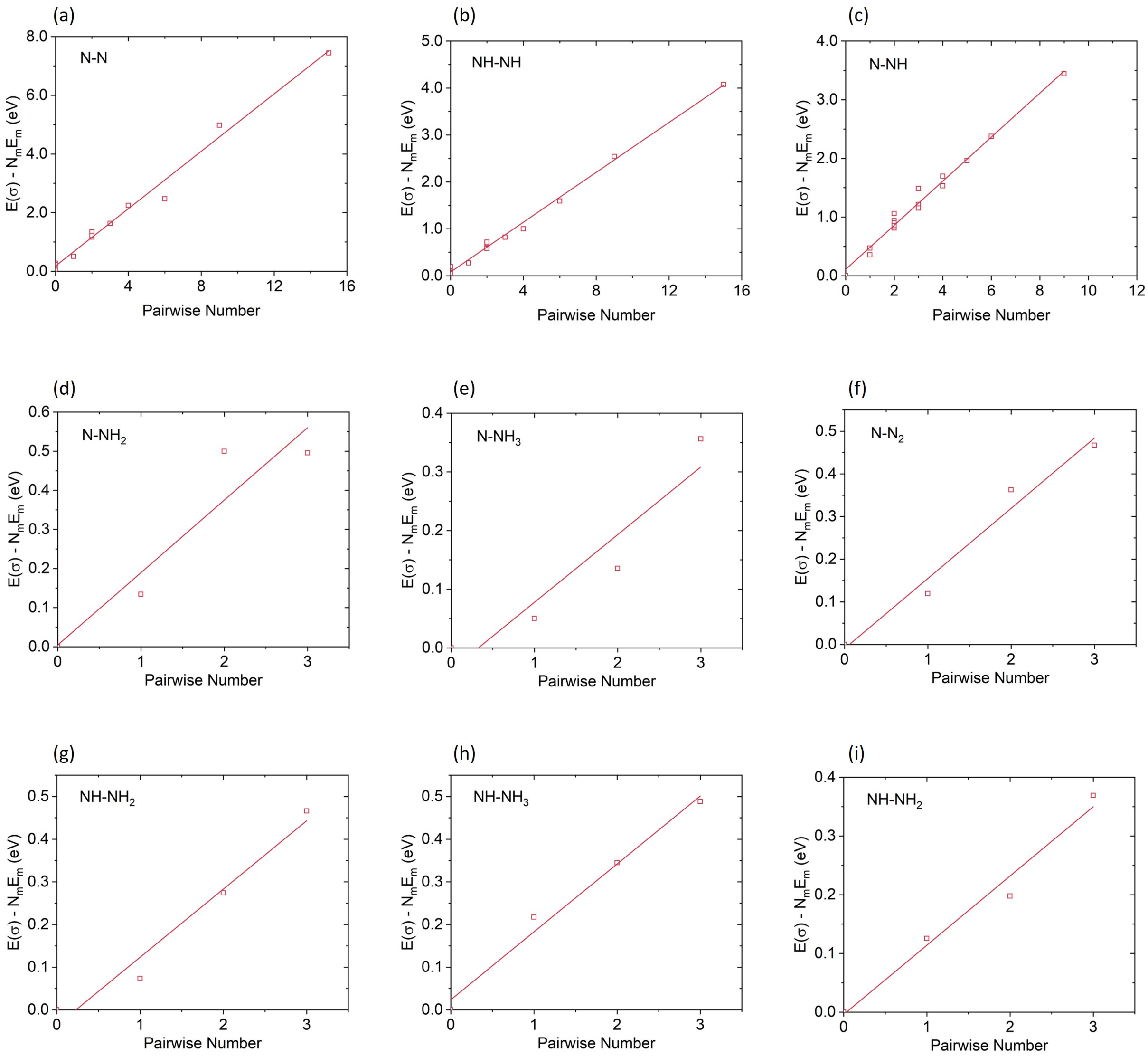

Supplementary Figure 10: Adsorbate-adsorbate interactions of majority species N and NH.

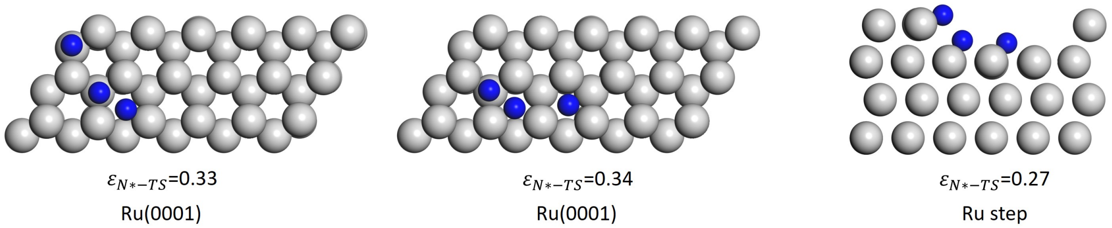

Supplementary Figure 11: Adsorbate-adsorbate interactions between N_2 dissociation transition state and N atom on Ru(0001) and a stepped Ru(0001) surfaces.

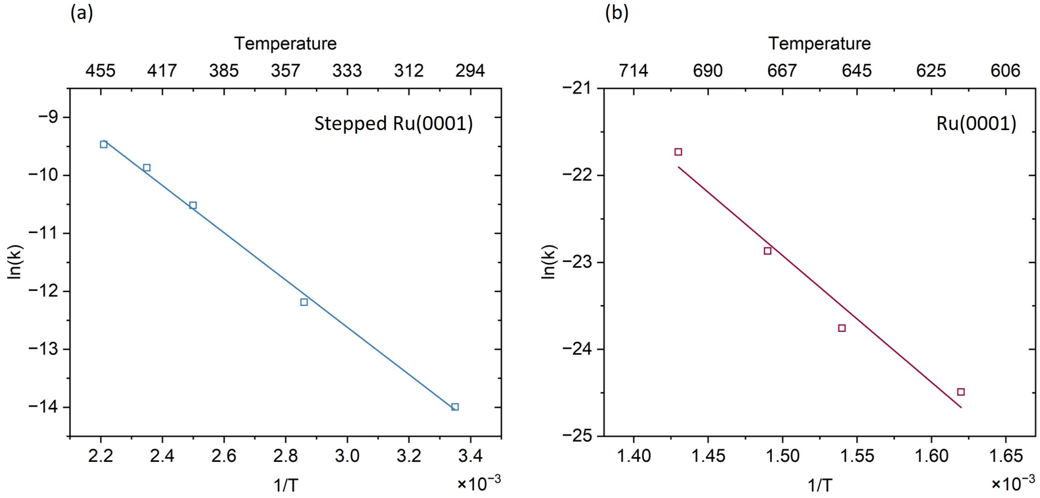

Supplementary Figure 12: Rate constant k versus temperatures calculated on (a) a stepped Ru(0001) surface and (b) Ru(0001).

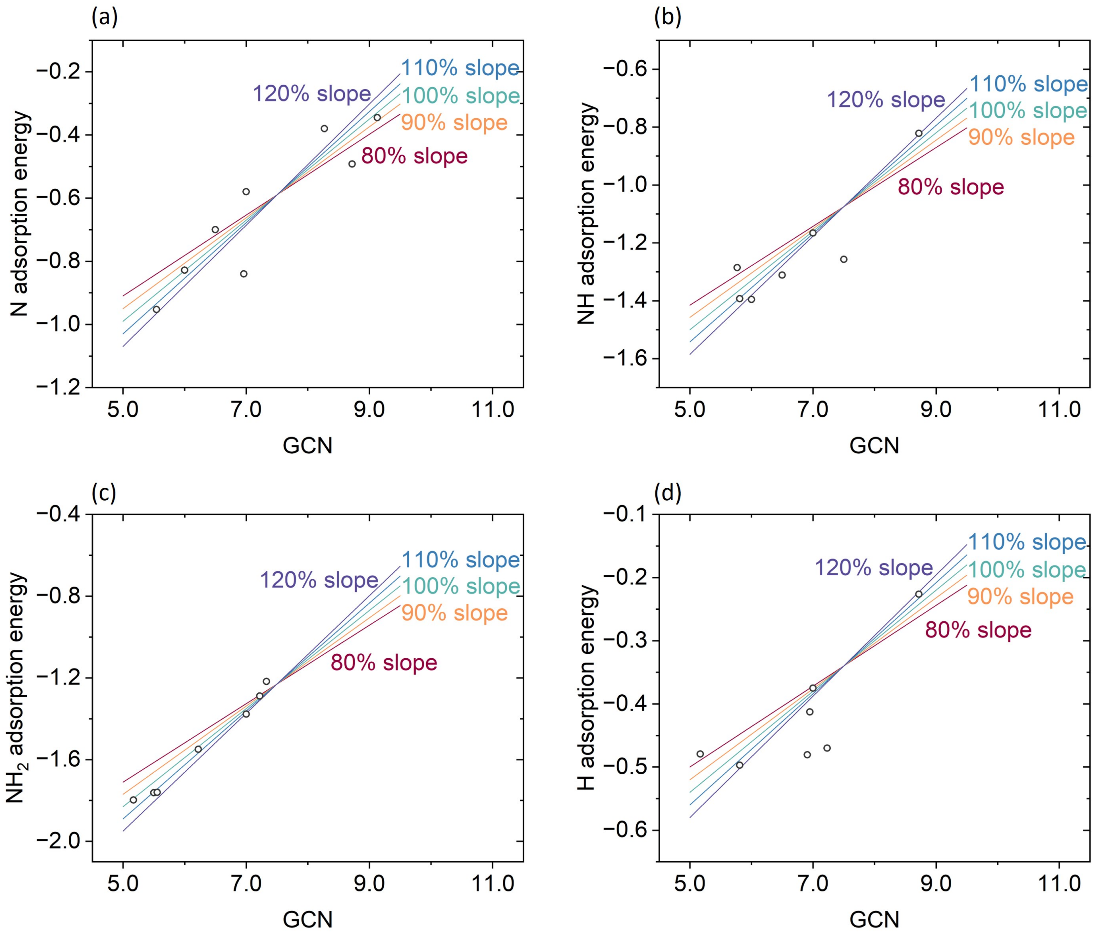

Supplementary Figure 13: Linear scaling relations with different slopes in uncertainty analysis

Supplementary Table 1: GCN and adsorption sites for the N atom as included in the fits in Figure 1a.

Surface	Adsorption site	GCN	Adsorption energy (eV)
Ru(0001)	hcp hollow	7.50	-0.98
Ru(10$\bar{1}$1)	4-fold hollow	6.96	-0.84
Ru(10$\bar{1}$0)	hcp hollow	7.00	-0.58
Ru(10$\bar{1}$2)	4-fold hollow	6.00	-0.83
Ru(2$\bar{1}\bar{1}$2)	hcp hollow	6.50	-0.70
Ru A step	hcp hollow	9.13	-0.35
Ru B step	hcp hollow	8.72	-0.49
Ru D step	hcp hollow	8.27	-0.38
Ru B edge	hcp hollow	5.55	-0.95

Supplementary Table 2: GCN and adsorption site for NH as included in the fits of Figure 1a.

Surface	Adsorption site	GCN	Adsorption energy (eV)
Ru(0001)	hcp hollow	7.50	-1.26
Ru(10$\bar{1}$1)	fcc hollow	5.81	-1.39
Ru(10$\bar{1}$0)	hcp hollow	7.00	-1.17
Ru(10$\bar{1}$2)	4-fold hollow	6.00	-1.40
Ru(2$\bar{1}\bar{1}$2)	hcp hollow	6.50	-1.31
Ru B step	hcp hollow	8.72	-0.82
Ru B edge	4-fold hollow	5.77	-1.29

Supplementary Table 3: GCN and adsorption site for NH_2 as included in the fits of Figure 1a.

Surface	Adsorption site	GCN	Adsorption energy (eV)
Ru(0001)	bridge	7.33	-1.22
Ru($10\bar{1}1$)	bridge	7.00	-1.38
Ru($10\bar{1}0$)	bridge	6.22	-1.55
Ru($10\bar{1}2$)	bridge	5.50	-1.76
Ru($2\bar{1}\bar{1}2$)	bridge	5.56	-1.76
Ru B step	bridge	7.22	-1.29
Ru B edge	bridge	5.17	-1.80

Supplementary Table 4: GCN and adsorption site for NH_3 as included in the fits of Figure 1a.

Surface	Adsorption site	GCN	Adsorption energy (eV)
Ru(0001)	top	7.50	-1.67
Ru($10\bar{1}1$)	top	6.83	-1.72
Ru($10\bar{1}0$)	top	6.67	-1.79
Ru($10\bar{1}2$)	top	5.67	-1.77
Ru($2\bar{1}\bar{1}2$)	top	5.67	-1.69
Ru B step	top	5.50	-1.79
Ru B edge	top	5.33	-1.77

Supplementary Table 5: GCN and adsorption site for H as included in the fits of Figure 1a.

Surface	Adsorption site	GCN	Adsorption energy (eV)
Ru(0001)	fcc hollow	6.95	-0.41
Ru(10$\bar{1}$1)	fcc hollow	5.81	-0.50
Ru(10$\bar{1}$0)	hcp hollow	7.00	-0.37
Ru(10$\bar{1}$2)	fcc hollow	6.91	-0.48
Ru(2$\bar{1}\bar{1}$2)	4-fold hollow	7.23	-0.47
Ru B step	hcp hollow	8.72	-0.23
Ru B edge	bridge	5.17	-0.48

Supplementary Table 6: GCN and adsorption site for N_2 as included in the fits of Figure 1a.

Surface	Adsorption site	GCN	Adsorption energy (eV)
Ru(0001)	top	7.50	-0.55
Ru(10$\bar{1}$1)	top	6.83	-0.62
Ru(10$\bar{1}$0)	top	6.67	-0.76
Ru(10$\bar{1}$2)	top	5.67	-0.76
Ru(2$\bar{1}\bar{1}$2)	top	5.67	-0.64
Ru B step	top	5.50	-0.75
Ru B edge	top	5.33	-0.76

Supplementary Table 7: N-adsorption energy (eV) calculated with different exchange-correlation functionals.

Surface	GCN	Exchange-correlation functionals			
		BEEF-vdW	PBE	PBE+D3	RPBE
Ru B edge	5.55	-0.95	-1.16	-1.30	-0.92
Ru($10\bar{1}2$)	6.00	-0.83	-1.08	-1.28	-0.80
Ru($2\bar{1}\bar{1}2$)	6.50	-0.70	-0.99	-1.11	-0.66
Ru($10\bar{1}1$)	6.96	-0.84	-1.07	-1.27	-0.78
Ru($10\bar{1}0$)	7.00	-0.58	-0.79	-0.99	-0.51
Ru(0001)	7.50	-0.98	-1.16	-1.33	-0.89
Ru D step	8.27	-0.38	-0.56	-1.02	-0.27
Ru B step	8.72	-0.49	-0.67	-0.89	-0.38
Ru A step	9.13	-0.35	-0.32	-0.53	-0.03

Supplementary Table 8: Surface energy on Wulff construction Ru NPs.

Ru surface	Surface energy (meV/Å^2)
Ru(0001)	165
Ru($10\bar{1}0$)	181
Ru($10\bar{1}1$)	180
Ru($10\bar{1}2$)	190
Ru($2\bar{1}\bar{1}2$)	194

Supplementary Table 9: Coverages obtained in kMC simulations compared to experimental[12] coverages of N, NH and NH_2 on Ru($10\bar{1}3$) surface.

Surface species		T=523 K p=0.5 bar	T=623 K p=0.5 bar	T=623 K p=0.2 bar
N*	kMC	0.52%	0.44%	0.58%
	Exp.	0.50%	/	/
NH*	kMC	1.37%	0.33%	0.32%
	Exp.	/	<0.10%	<0.10%
NH_2*	kMC	0.04%	0.09%	0.08%
	Exp.	0.10%	<0.10%	<0.10%

Supplementary Table 10: Compilation of experimental TOF and apparent activation energies (E_a^{app}).

Catalysts	Temperature (K)	Pressure (MPa)	TOF ($site^{-1}$ s^{-1})	E_a^{app} (eV)	Reference
Ru/C	673	1.0	0.036	0.99	Li et al.[13]
Ru-Ba/C	673	1.0	0.020	0.93	Kitano et al.[14]
Ru/CeO_2	663	0.9	0.014	/	Sato et al.[15]
Ru/MgO	663	0.9	0.003	/	Sato et al.[15]
Ru/ZSM-5	673	1.0	0.006	0.61	Li et al.[16]
Ru/BHA	653	1.1	0.0071	/	You et al.[17]
Ru/MgO	653	1.1	0.0021	/	You et al.[17]
Ru/Ti-Ce-S	673	1.0	0.0141	0.79	Wu et al.[18]

Supplementary Table 11: Parameters in the linear scaling relations with the descriptor of GCN.

Surface species	$\Delta E = c \cdot GCN + d$	
	c	d
N	0.16	-1.79
NH	0.17	-2.35
NH_2	0.24	-3.03
NH_3	0.03	-1.91
H	0.08	-0.94
N_2	0.07	-1.13

Supplementary Table 12: Parameters used in the linear scaling relations of barriers (E_a) with respect to reaction energies (ΔE).

Elementary reaction	$E_a = a\Delta E + b$	
	a	b
${N_2}^* + * \rightleftharpoons 2N^*$	-0.38	1.00
$N^* + H^* \rightleftharpoons NH^* + *$	-1.23	1.31
$NH^* + H^* \rightleftharpoons {NH_2}^* + *$	0.15	1.33
${NH_2}^* + H^* \rightleftharpoons {NH_3}^* + *$	0.26	1.35

Supplementary Table 13: Reaction energy and energy barrier for ${N_2}^* + {}^* \rightleftharpoons 2N^*$ in the fits in Figure 1a.

Surface	Reaction energy (eV)	Energy barrier (eV)
Ru(0001)	-1.38	1.73
Ru(10$\bar{1}$1)	-1.06	1.12
Ru(10$\bar{1}$0)	-0.40	1.34
Ru(10$\bar{1}$2)	-0.90	1.22
Ru(2$\bar{1}\bar{1}$2)	-0.76	1.41
Ru A step	0.04	1.06
Ru B step	-0.23	0.91

Supplementary Table 14: Reaction energy and energy barrier for $N^* + H^* \rightleftharpoons NH^* + {}^*$ in the fits in Supplementary Figure 1.

Surface	Reaction energy (eV)	Energy barrier (eV)
Ru(0001)	0.12	1.45
Ru(10$\bar{1}$1)	-0.06	1.33
Ru(10$\bar{1}$0)	-0.21	1.12
Ru(10$\bar{1}$2)	-0.09	1.21
Ru(2$\bar{1}\bar{1}$2)	-0.14	1.03
Ru B step	-0.10	1.12

Supplementary Table 15: Reaction energy and energy barrier for $NH^* + H^* \rightleftharpoons NH_2{}^* + {}^*$ in the fits in Supplementary Figure 1.

Surface	Reaction energy (eV)	Energy barrier (eV)
Ru(0001)	0.45	1.43
Ru(10$\bar{1}$1)	0.51	1.36
Ru(10$\bar{1}$0)	-0.01	1.25
Ru(10$\bar{1}$2)	0.11	1.29
Ru(2$\bar{1}\bar{1}$2)	0.02	1.46
Ru B step	-0.24	1.28

Supplementary Table 16: Reaction energy and energy barrier for $NH_2{}^* + H^* \rightleftharpoons NH_3{}^* + {}^*$ in the fits in Supplementary Figure 1.

Surface	Reaction energy (eV)	Energy barrier (eV)
Ru(0001)	-0.04	1.37
Ru(10$\bar{1}$1)	0.15	1.50
Ru(10$\bar{1}$0)	0.14	1.29
Ru(10$\bar{1}$2)	0.47	1.46
Ru(2$\bar{1}\bar{1}$2)	0.54	1.49
Ru B step	-0.27	1.27

Supplementary Table 17: ZPE corrections and entropy corrections at 673 K.

Gas/surface species	ZPE(eV)	Entropy (eV/K) $\times 10^{-3}$
N_2(g)	0.16	2.24
H_2(g)	0.30	1.60
NH_3(g)	0.94	2.34
N_2*	0.20	0.81
N*	0.08	0.30
H*	0.16	0.16
NH*	0.38	0.42
NH_2*	0.70	0.63
NH_3*	1.02	0.99
TS(N-N*)	0.12	0.53
TS(N-H*)	0.19	0.42
TS(NH-H*)	0.49	0.58
TS(NH_2-H*)	0.84	0.79

Supplementary Table 18: Parameters in the linear scaling relations with the descriptor of strain (Δs).

Elementary reaction	$E_a^{strain} = E_a + \alpha\Delta s$	$E^{strain} = \Delta E + \beta\Delta s$
	α	β
$N_2(g) + * \rightleftharpoons {N_2}^*$	-	-1.58
$H_2(g) + * \rightleftharpoons 2H^*$	-	-4.50
${N_2}^* + * \rightleftharpoons 2N^*$	-4.44	-11.83
$N^* + H^* \rightleftharpoons NH^* + *$	4.59	3.97
$NH^* + H^* \rightleftharpoons {NH_2}^* + *$	1.50	3.57
${NH_2}^* + H^* \rightleftharpoons {NH_3}^* + *$	2.89	4.51
${NH_3}^* \rightleftharpoons NH_3(g)$	-	-1.42

Supplementary Table 19: Reaction energies on +4% and -4% strained Ru(0001) and stepped Ru(0001) surfaces. The energies are with respect to unstrained surfaces.

Elementary reaction	Ru(0001)		Stepped Ru(0001)	
	-4%	+4%	-4%	+4%
$N_2(g) + * \rightleftharpoons {N_2}^*$	0.09	-0.09	0.03	-0.03
$H_2(g) + * \rightleftharpoons 2H^*$	0.21	-0.11	0.24	-0.16
${N_2}^* + * \rightleftharpoons 2N^*$	0.56	-0.32	0.47	-0.55
$N^* + H^* \rightleftharpoons NH^* + *$	-0.27	0.16	-0.08	0.13
$NH^* + H^* \rightleftharpoons {NH_2}^* + *$	-0.18	0.06	-0.20	0.13
${NH_2}^* + H^* \rightleftharpoons {NH_3}^* + *$	-0.12	0.07	-0.28	0.25
${NH_3}^* \rightleftharpoons NH_3(g)$	0.09	-0.07	0.05	-0.02

Supplementary Table 20: Energy barriers on +4% and -4% strained Ru(0001) and stepped Ru(0001) surfaces. The energies are with respect to unstrained surfaces.

Elementary reaction	Ru(0001)		Stepped Ru(0001)	
	-4%	+4%	-4%	+4%
${N_2}^* + * \rightleftharpoons 2N^*$	0.18	-0.11	0.28	-0.15
$N^* + H^* \rightleftharpoons NH^* + *$	-0.14	0.08	-0.18	0.34
$NH^* + H^* \rightleftharpoons {NH_2}^* + *$	-0.18	-0.03	-0.05	0.05
${NH_2}^* + H^* \rightleftharpoons {NH_3}^* + *$	-0.06	0.05	-0.12	0.07

Supplementary Table 21: Pairwise adsorbate-adsorbate interactions (eV).

	*	N*	NH*	NH_2*	NH_3*	N_2*	H*
*	0	0	0	0	0	0	0
N*	0	0.49	0.38	0.19	0.12	0.16	0
NH*	0	0.38	0.27	0.16	0.16	0.12	0
NH_2*	0	0.19	0.16	0.08	0.07	0.07	0
NH_3*	0	0.12	0.16	0.07	0.06	0.05	0
N_2*	0	0.16	0.12	0.07	0.05	0.05	0
H*	0	0	0	0	0	0	0

Supplementary Table 22: Parameters in the calculations of scaling factor κ at 673 K.

	Ru(0001)	Stepped Ru(0001)
q_{TS}	4.60×10^{2}	4.60×10^{2}
q_{gas}	1.84×10^{11}	1.84×10^{11}
ΔG_{act}	1.30	0.4
$k_{collision}$	1.36×10^{-10}	1.59×10^{-3}
κ	2.11×10^{-5}	4.49×10^{-5}

Supplementary Table 23: Relative TOF difference (ΔTOF) by each surface species. Combined effect represents adjusting the scaling relations for N, NH, NH_2 and NH_3 simultaneously.

	Slope changes				
	-20%	-10%	0	10%	20%
NH	4.82%	2.58%	0	4.00%	1.43%
NH_2	0.47%	3.58%	0	5.05%	5.23%
H	15.33%	7.33%	0	1.92%	-3.91%
N	26.19%	14.65%	0	-8.87%	-16.43%
Combined effect	16.98%	8.67%	0	-9.65%	-13.46%

Supplementary Table 24: TOF with respect to strain in Figure 2a ($site^{-1}$ s^{-1}).

Strain	$\sigma = 0$	$\sigma = 0.05$
-0.04	1.26×10^{-5}	4.23×10^{-4}
-0.02	6.53×10^{-5}	1.35×10^{-3}
0	3.33×10^{-4}	2.84×10^{-3}
0.02	1.24×10^{-3}	3.82×10^{-3}
0.04	1.79×10^{-3}	3.70×10^{-3}
0.06	1.52×10^{-3}	2.49×10^{-3}
0.08	6.58×10^{-4}	1.38×10^{-3}
0.10	3.17×10^{-4}	6.22×10^{-4}
0.12	6.29×10^{-5}	2.06×10^{-4}

Supplementary Table 25: TOF by different energy parameters on Ru(0001) surface ($site^{-1}$ s^{-1}).

Catalysts	Linear scaling relations	Explicit DFT energy
Stepped Ru(0001) surface	7.0×10^{-5}	6.7×10^{-4}
Ru_{780} NP	3.3×10^{-4}	3.0×10^{-4}

Supplementary References

(1) Hellwege, K.; Hellwege, A.; Eisenmann, B.; Schaefer, H. *Structure data of elements and intermetallic phases*; Springer, 1971; Vol. 3.

(2) Henkelman, G.; Uberuaga, B. P.; Jónsson, H. A climbing image nudged elastic band method for finding saddle points and minimum energy paths. *J. Chem. Phys.* **2000**, *113*, 9901–9904.

(3) NIST Chemistry WebBook. `https://doi.org/10.18434/T4D303`.

(4) Jørgensen, M.; Gronbeck, H. Adsorbate entropies with complete potential energy sampling in microkinetic modeling. *J. Phys. Chem. C* **2017**, *121*, 7199–7207.

(5) Dietze, E. M.; Grönbeck, H. Structure-dependent strain effects. *ChemPhysChem* **2020**, *21*, 2407–2410.

(6) Honkala, K.; Hellman, A.; Remediakis, I.; Logadottir, A.; Carlsson, A.; Dahl, S.; Christensen, C. H.; Nørskov, J. K. Ammonia synthesis from first-principles calculations. *Science* **2005**, *307*, 555–558.

(7) Nørskov, J. K.; Bligaard, T.; Logadottir, A.; Bahn, S.; Hansen, L. B.; Bollinger, M.; Bengaard, H.; Hammer, B.; Sljivancanin, Z.; Mavrikakis, M.; others Universality in heterogeneous catalysis. *J. Catal.* **2002**, *209*, 275–278.

(8) Dahl, S.; Logadottir, A.; Egeberg, R.; Larsen, J.; Chorkendorff, I.; Törnqvist, E.; Nørskov, J. K. Role of steps in N2 activation on Ru(0001). *Phys. Rev. Lett.* **1999**, *83*, 1814.

(9) Stegelmann, C.; Andreasen, A.; Campbell, C. T. Degree of rate control: how much the energies of intermediates and transition states control rates. *J. Am. Chem. Soc.* **2009**, *131*, 8077–8082.

(10) Meskine, H.; Matera, S.; Scheffler, M.; Reuter, K.; Metiu, H. Examination of the concept of degree of rate control by first-principles kinetic Monte Carlo simulations. *Surf. Sci.* **2009**, *603*, 1724–1730.

(11) Abild-Pedersen, F.; Greeley, J.; Studt, F.; Rossmeisl, J.; Munter, T. R.; Moses, P. G.; Skulason, E.; Bligaard, T.; Nørskov, J. K. Scaling Properties of Adsorption Energies for Hydrogen-Containing Molecules on Transition-Metal Surfaces. *Phys. Rev. Lett.* **2007**, *99*, 016105.

(12) Goodwin, C. M.; Lömker, P.; Degerman, D.; Davies, B.; Shipilin, M.; Garcia-Martinez, F.; Koroidov, S.; Katja Mathiesen, J.; Rameshan, R.; Rodrigues, G. L.; others Operando probing of the surface chemistry during the Haber-Bosch process. *Nature* **2024**, *625*, 282–286.

(13) Li, L.; Jiang, Y.-F.; Zhang, T.; Cai, H.; Zhou, Y.; Lin, B.; Lin, X.; Zheng, Y.; Zheng, L.; Wang, X.; others Size sensitivity of supported Ru catalysts for ammonia synthesis: From nanoparticles to subnanometric clusters and atomic clusters. *Chem* **2022**, *8*, 749–768.

(14) Kitano, M.; Inoue, Y.; Yamazaki, Y.; Hayashi, F.; Kanbara, S.; Matsuishi, S.; Yokoyama, T.; Kim, S.-W.; Hara, M.; Hosono, H. Ammonia synthesis using a stable electride as an electron donor and reversible hydrogen store. *Nat. chem.* **2012**, *4*, 934–940.

(15) Sato, K.; Imamura, K.; Kawano, Y.; Miyahara, S.-i.; Yamamoto, T.; Matsumura, S.; Nagaoka, K. A low-crystalline ruthenium nano-layer supported on praseodymium oxide as an active catalyst for ammonia synthesis. *Chem. Sci.* **2017**, *8*, 674–679.

(16) Li, L.; Cai, J.; Liu, Y.; Ni, J.; Lin, B.; Wang, X.; Au, C.-t.; Jiang, L. Zeolite-seed-directed Ru nanoparticles highly resistant against sintering for efficient nitrogen activation to ammonia. *Sci. Bull.* **2020**, *65*, 1085–1093.

(17) You, Z.; Inazu, K.; Aika, K.-i.; Baba, T. Electronic and structural promotion of barium hexaaluminate as a ruthenium catalyst support for ammonia synthesis. *J. Catal.* **2007**, *251*, 321–331.

(18) Wu, Y.; Li, C.; Fang, B.; Wang, X.; Ni, J.; Lin, B.; Lin, J.; Jiang, L. Enhanced ammonia synthesis performance of ceria-supported Ru catalysts via introduction of titanium. *Chem. Commun.* **2020**, *56*, 1141–1144.
